# Construction and Evaluation of Hepatic Targeted Drug Delivery System with Hydroxycamptothecin in Stem Cell-Derived Exosomes

**DOI:** 10.3390/molecules29215174

**Published:** 2024-10-31

**Authors:** Qiongjun Zhao, Zixuan Mo, Liuting Zeng, Yue Yuan, Yan Wang, Ying Wang

**Affiliations:** 1 School of Chinese Materia Medica, Guangdong Pharmaceutical University, Guangzhou 510006, China; zhaoqiongjun3647@163.com (Q.Z.); mozixuan7908@163.com (Z.M.); zliuting0104@163.com (L.Z.); yuanyue2105@163.com (Y.Y.); 2 Teaching and Experimental Center, Guangdong Pharmaceutical University, Zhongshan 528453, China

**Keywords:** exosomes, hydroxycamptothecin, SP94 targeting peptide, hepatocellular carcinoma

## Abstract

Hydroxycamptothecin (HCPT) is commonly used in the treatment of liver cancer; however, its low water solubility and poor stability significantly limit its clinical application. In recent years, research on exosomes has deepened considerably. Exosomes possess a unique phospholipid bilayer structure, enabling them to traverse tissue barriers, which provides natural advantages as drug carriers. Nevertheless, delivering exosomes safely and efficiently to target cells remains a major challenge. In this study, we utilized the affinity of the SP94 peptide for human liver cancer cell receptors. HCPT was coated with exosomes in our experimental design, and the exosome membrane was modified with SP94 peptide to facilitate drug delivery to liver cancer cells. Exosomes were purified from bone marrow mesenchymal stem cells, and targeted peptides were attached to their surfaces via post-insertion techniques. Subsequently, HCPT was incorporated into the exosomes through electroporation. Using the HepG2 hepatoma cell line, we evaluated a series of in vitro pharmacodynamics and studied pharmacokinetics and tissue distribution in animal models. The results indicated that ligand-targeted, modified drug-carrying exosomes significantly enhance drug bioavailability, prolong retention time in vivo, and facilitate liver targeting. Moreover, this approach reduces drug nephrotoxicity, enhances anti-tumor efficacy, and lays the groundwork for the development of novel liver cancer-targeting agents.

## 1. Introduction

Hepatocellular carcinoma (HCC) is the most common malignant tumor in primary liver cancer, and its incidence is on the rise worldwide. Hydroxycamptothecin (HCPT) is a kind of DNA synthesis inhibitor, and its mechanism of action is mainly through acting on the S phase of the cell cycle, binding with DNA topoisomerase I (Topo I), interfering with the processes of DNA replication, transcription, and repair, causing the apoptosis of tumor cells, and ultimately achieving the purpose of anti-tumor [1]. However, HCPT is limited by its poor water solubility, low bioavailability, and fast metabolism in vivo, which hinder its therapeutic effectiveness. Consequently, there is a critical need to develop novel nanodrug delivery systems to enhance the clinical application of small-molecule drugs.

Exosomes are extracellular vesicles secreted by cells, ranging from 40 to 160 nm in size, with a phospholipid bilayer containing various bioactive components. They function as mediators of intercellular communication and substance exchange [2,3]. Exosomes are heterogeneous, with their contents varying depending on their cellular origin. They have been reported to carry a variety of bioactive molecules, including nucleic acids, proteins, and lipids. Nucleic acids in exosomes, such as DNA, mRNA, and siRNA, are known to stably express proteins like CD9, CD63, TSG101, and Alix and are involved in regulating a multitude of physiological activities. These activities include tissue repair, angiogenesis, cell metabolism, and immune responses [4,5,6,7,8,9]. Almost all living cells can produce exosomes, with mesenchymal stem cells (MSCs) being a particularly noteworthy source. MSCs are pluripotent stem cells with self-renewal capabilities, multi-lineage differentiation potential, and immunomodulatory properties, enabling them to regulate various immune response-related cells [10,11]. The therapeutic effects of stem cells are often attributed to paracrine mechanisms, which are crucial for maintaining intracellular environmental balance and intercellular communication [12,13]. Exosomes play a pivotal role in these paracrine effects [14]. The exosomes secreted by MSCs (MSCs-Exos) also have a similar immunomodulatory mechanism, which can alleviate inflammation and immune response [15,16]. Compared with original cells, MSCS-derived exosomes not only have similar biological functions but also lower immunogenicity and better stability, so MSCS-derived exosomes become a substitute for cell therapy. MSCs-Exos have been shown to inhibit inflammatory responses in various settings by inducing M2 macrophage polarization and regulating T cells [17,18].

Although natural exosomes have cup-shaped bilayer membrane structures and surface proteins, which can effectively evade the body’s immune system, promote tumor vascular extravasation, and target diseased tissues, unmodified drug carriers still have poor targeting ability and the potential loss of drug cargo during transport [19,20]. Therefore, it is necessary to develop engineered exosomes; that is, the surface of exosomes is modified by targeting. Recent studies have shown that DSPE-PEG2000 can bind to molecules such as c-Met, RGD, folic acid, and streptavidin. When targeting ligands are coupled to nanocarriers, they facilitate receptor-mediated internalization, improve the targeting efficiency of the carrier, and enhance therapeutic outcomes. Moreover, after modification, it can prolong the in vivo circulation time of the drug-carrying system and target the tumor through the EPR effect [21,22]. Researchers have identified a novel peptide, SP94 (SFSIIHTPILPL), using phage display peptide libraries. The SP94 peptide has been shown to have a high affinity for receptors expressed on human liver cancer cells [23]. It can bind specifically to hepatocellular carcinoma cells in vitro or in vivo to enhance the therapeutic effect of hepatocellular carcinoma xenotransplantation by enhancing tumor cell apoptosis and reducing tumor angiogenesis. Thus, the SP94 peptide holds clinical potential for improving the systemic treatment of advanced hepatocellular carcinoma.

In this study, exosomes derived from mesenchymal stem cells were used as drug carriers. HCPT was encapsulated in exosomes, and the targeting peptides were conjugated. Exosomes were characterized, and the release curves of the preparations in vitro were evaluated in different release media. In addition, a series of in vitro pharmacodynamic evaluation, pharmacokinetics, and tissue distribution studies were conducted.

## 2. Results

### 2.1. Identification and Characterization of Exosomes

**Figure 1:**
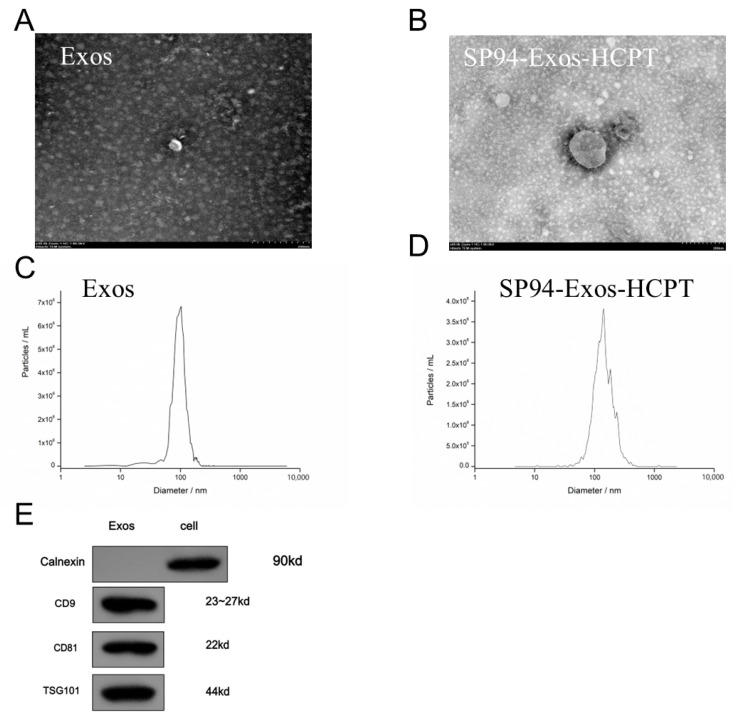
Characterization of exosomes. (**A**) TEM detection of the blank exosomes (Exos) morphology (Bar = 200 nm). (**B**) TEM image of drug-loaded exosomes (SP94-Exos−HCPT). (**C**) Size distributions of Exos. The mean particle size was 103 nm. (**D**) Size distributions of SP94-Exos-HCPT. The mean particle size was 133 nm. The data from C and D were based on the NTA measurements. (**E**) Exosomal biomarkers of Exos were detected by the Western blot analysis.

Exosomes purified from the supernatant of mesenchymal stem cells were characterized using transmission electron microscopy (TEM) and nano particle analyzer (NTA). TEM images confirmed that both Exos and SP94-Exos-HCPT appear as oval structures with clear edges, indicating that the exosome structure remained intact before and after drug loading (Figure 1A,B). NTA confirmed that the average particle size of Exos was 103.1 nm. Following targeted modification and drug loading, the average particle size of the exosomes increased slightly to 133.5 nm (Figure 1C,D). Western blot analysis demonstrated clear bands for the exosome-specific proteins CD9, CD81, and TSG101 (Figure 1E), while Calnexin, a specific protein of the endoplasmic reticulum, was not detected in the exosomes. These results indicate that the extracted exosomes were of high quality and suitable for subsequent experiments.

### 2.2. In Vitro Drug Release

**Figure 2:**
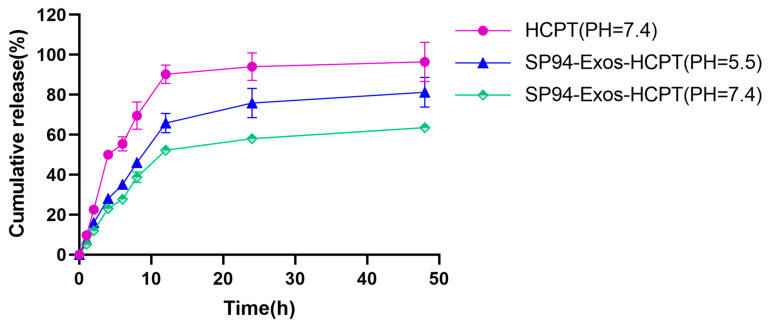
In vitro release profiles of HCPT and SP94-Exos−HCPT (error bars represent means ± standard deviation (SD) from three independent replicates).

The drug release profiles of exosomes in dissolution media at pH 7.4 and pH 5.5 were evaluated using dynamic dialysis. As shown in Figure 2, at pH 7.4, the free drug exhibited rapid release within the first 6 h, with a cumulative release reaching 94.0% at 24 h. In contrast, under the same conditions, SP94-Exos-HCPT demonstrated a cumulative release of 58.0% at 24 h, indicating that SP94-Exos-HCPT possesses sustained-release properties in a physiological environment. This suggested that the exosome membrane effectively retained the drug. However, in the release medium with a pH of 5.5, which simulated the tumor cell environment, SP94-Exos-HCPT exhibited a relatively rapid release, with a cumulative release rate of 75.8% after 24 h. This suggested that the exosome membrane structure was more prone to disruption in acidic environments, leading to enhanced membrane fluidity and accelerated drug release.

### 2.3. Cell Proliferation Assay (CCK-8 Method)

To determine the appropriate concentration of HCPT, a cytotoxicity assay was performed using the CCK-8 method. The IC50 value of HCPT for HepG2 cells was 23.72 μg/mL; therefore, a concentration of 20 μg/mL was selected for subsequent experiments.

**Figure 3:**
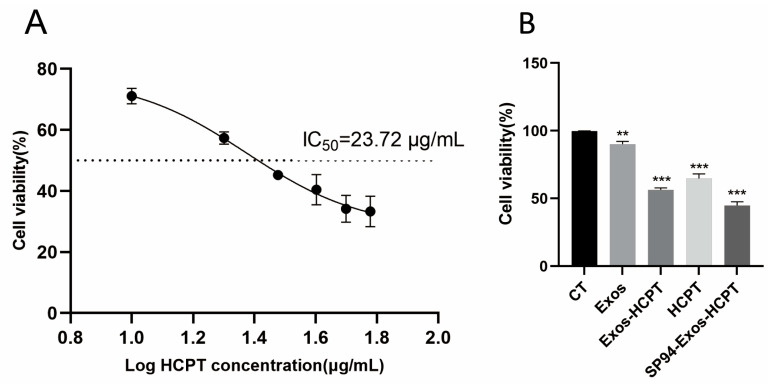
(**A**) The influence of HCPT on HepG2 cell vitality. (**B**) Effects of different drug groups on the proliferation of HepG2 cells in vitro (error bars represent means ± standard deviation (SD) from three independent replicates; ** *p* < 0.01, *** *p* < 0.001).

The inhibitory effect of SP94-Exos-HCPT on the proliferation of HepG2 cells was also evaluated using the CCK-8 method. As shown in Figure 3, compared with the untreated control group, the survival rates of HepG2 cells treated with blank exosomes, Exos-HCPT, HCPT, and SP94-Exos-HCPT were reduced by 9.76%, 43.58%, 34.99%, and 55.00%, respectively, all of which were statistically significant. Notably, the Exos-HCPT group demonstrated stronger inhibition of HepG2 cell proliferation compared with the HCPT group, likely due to the enhanced cellular uptake of exosomes, thereby improving drug efficacy. Furthermore, the SP94-Exos-HCPT group exhibited superior inhibition of HepG2 cell proliferation compared with both the HCPT and Exos-HCPT groups, suggesting that the SP94-targeted peptide enhances drug delivery efficiency.

### 2.4. Cell Migration and Invasion

**Figure 4:**
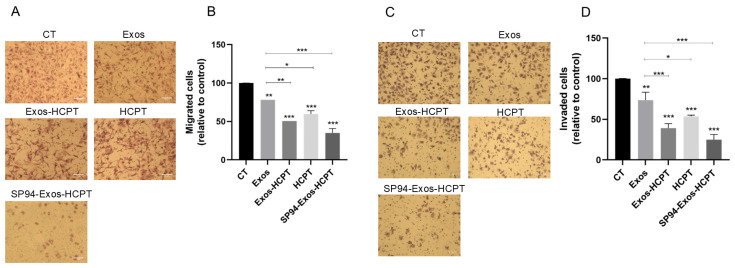
Cell migration and invasion were observed using a microscope, and the inhibition rate was calculated. (**A**) Representative diagram of cell migration. (**B**) The inhibition rate of cell migration. (**C**) Representative diagram of cell invasion. (**D**) The inhibition rate of cell invasion. (Drug group with 20 μg/mL Exos−HCPT, 20 μg/mL HCPT, and 20 μg/mL SP94-Exos−HCPT, Bar = 75 μm Error bars represent means ± standard deviation (SD) from three independent replicates. * *p* < 0.05, ** *p* < 0.01, *** *p* < 0.001).

Since the invasion and migration of cancer cells are closely related to tumor progression, the effects of different preparations on the migration and invasion of HepG2 cells were investigated using a Transwell assay. Figure 4A,C illustrate the migration and invasion behavior of HepG2 cells across the various drug groups. A decreasing trend in cell migration was observed in the Exos-HCPT, HCPT, and SP94-Exos-HCPT groups, with mobility rates of 50.61%, 59.65%, and 35.08%, respectively, showing statistically significant differences between the groups (Figure 4B). It was consistent with the result that SP94-Exos-HCPT significantly inhibited the invasion of HepG2 cells with the lowest invasion rate of 24.92% (Figure 4D). These findings suggest that SP94-Exos-HCPT effectively suppresses the migration and invasion of HepG2 cells, reducing both the penetration efficiency and chemotactic ability of HepG2 cells.

### 2.5. Cell Apoptosis and Cycle

**Figure 5:**
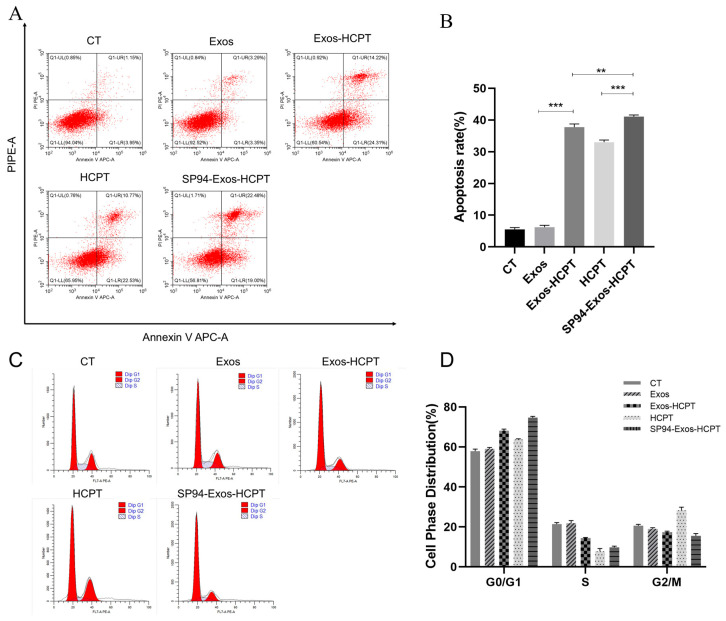
Flow Cytometry Analysis of the Cell Apoptosis and Cycle. (**A**) Apoptosis flow diagram of HepG2 cells treated with different preparations for 24 h. (**B**) The rate of cell apoptosis. (**C**) Flow diagram of cycle distribution of HepG2 cells after 24 h treatment with different preparations. (**D**) Cell cycle proportion in different groups. (drug group with 20 μg/mL Exos−HCPT, 20 μg/mL HCPT, and 20 μg/mL SP94-Exos−HCPT; error bars represent means ± standard deviation (SD) from three independent replicates. ** *p* < 0.01, *** *p* < 0.001).

Apoptosis was assessed using Annexin V-FITC double staining. The Exos treatment group exhibited minimal apoptosis and necrosis, with an apoptosis rate of 6.21%. In contrast, the apoptosis rates in the Exos-HCPT, HCPT, and SP94-Exos-HCPT groups were 37.76%, 33.01%, and 41.12%, respectively, indicating significantly higher rates of apoptosis and necrosis. These findings suggest that HCPT-Exos and SP94-targeted peptides can markedly promote tumor cell apoptosis. The highest apoptosis rate observed in the SP94-Exos-HCPT group may be attributed to the exosome bilayer lipid membrane structure, which facilitates cell communication, and the SP94 peptide’s ability to target the GRP78 receptor expressed in HepG2 cells [24], thereby enhancing apoptosis induction (Figure 5A,B).

Furthermore, HCPT induces apoptosis or necrosis in HepG2 cells by causing DNA damage. Compared with the control group, the proportion of cells in the G0/G1 phase increased in the Exos-HCPT and SP94-Exos-HCPT groups, while the number of cells in the S phase and G2/M phase decreased. The S phase corresponds to the synthesis of DNA and related histones, while the G2/M phase is the stage where DNA replication is completed and mitosis begins. In the HCPT, Exos-HCPT, and SP94-Exos-HCPT groups, the proportion of cells in S and G2/M phases was gradually reduced, indicating that these treatments can disrupt DNA synthesis and inhibit cell proliferation. The higher proportion of cells in the G0/G1 phase in the SP94-Exos-HCPT group suggests that this treatment alters cell cycle distribution, affects the mitotic process of tumor cells, and synergistically promotes apoptosis in HepG2 cells (Figure 5C,D).

### 2.6. Pharmacokinetic Studies

**Figure 6:**
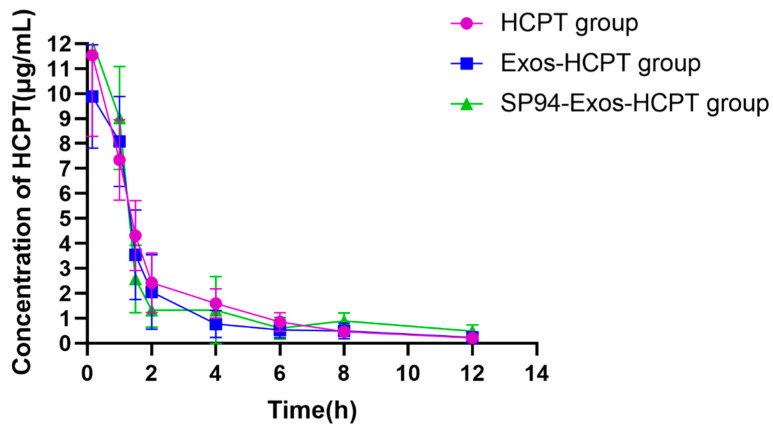
The concentration–time curves of HCPT in plasma of SD rats given HCPT Inject, Exos-HCPT, and SP94-Exos-HCPT after tail vein injection.

The pharmacokinetics of Exos-HCPT injection group and SP94-Exos-HCPT injection group were studied with HCPT injection group as the control. Following the tail vein in mice, all three groups of drugs exhibited rapid elimination from the bloodstream, with undetectable drug levels after 12 h, indicating a nonlinear distribution in vivo (Figure 6). Pharmacokinetic software DAS 2.0 was used for analysis, and the main pharmacokinetic parameters were obtained, as shown in Table 1. As can be seen from the table, the elimination half-life time (t_1/2_) of the HCPT injection group was approximately 2.45 h, while that of the SP94-Exos-HCPT injection group was about 5.3 h. This elimination half-life time in the SP94-Exos-HCPT group suggests prolonged drug efficacy, likely related to its drug-loading method. The mean residence time (MRT_(0-∞)_) for the SP94-Exos-HCPT injection group was 4.85 ± 3.08 h, which was higher than that of the HCPT injection group, whose MRT_(0-∞)_ was 2.77 ± 0.37 h. This indicates that after encapsulation in exosomes, the drug is eliminated more slowly from the body, resulting in a longer retention time. These findings suggest that HCPT is released more gradually when delivered via targeted exosomal systems, maintaining a relatively stable blood concentration and thereby extending the therapeutic duration of HCPT in vivo.

### 2.7. Tissue Distribution Studies

**Figure 7:**
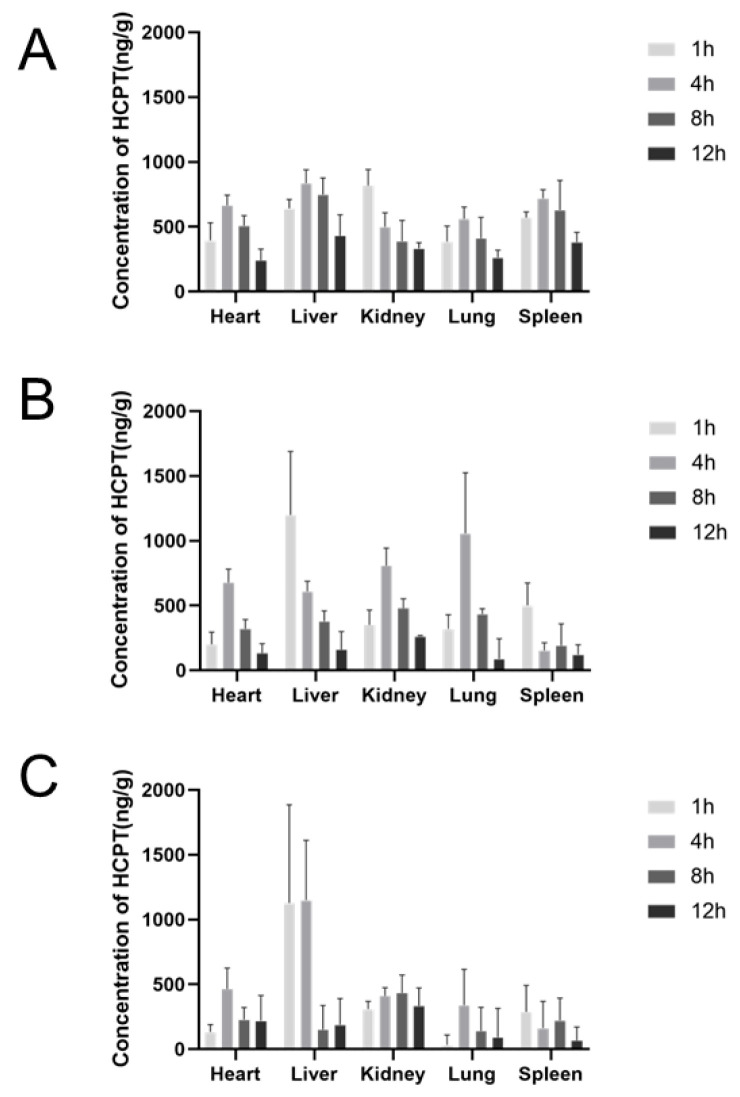
Tissue distribution of different preparations after intravenous injection (**A**) HCPT injection (**B**) Exos-HCPT injection (**C**) SP94-Exos-HCPT injection.

In the HCPT injection group, the highest concentration of HCPT in the spleen was observed 1 h post-administration. After 4 h, the drug predominantly accumulated in the liver and kidneys, indicating these organs have a high uptake capacity for HCPT. Over time, the drug concentration in all tissues gradually decreased (Figure 7A). Following intravenous injection of Exos-HCPT via the tail vein, HCPT rapidly distributed across all tissues, with peak concentrations in the liver and kidneys occurring 1 h post-administration. The tissue distribution hierarchy, from highest to lowest concentration, was liver > kidney > spleen > lung > heart, with the liver showing the highest concentration (Figure 7B). In the SP94-Exos-HCPT injection group, HCPT also rapidly distributed across tissues, reaching the highest concentration in the kidney 1 h after administration. By 4 h post-administration, the highest drug concentrations were found in the heart, liver, spleen, and lungs, with the concentration hierarchy being liver > heart > spleen > lung (Figure 7C).

Quantitative drug analysis in dissected tissues at specific time points post-administration revealed that all groups exhibited rapid absorption and widespread tissue distribution following tail vein injection. Across different administration groups, drug concentrations were consistently higher in the liver during the same time period, likely due to the liver’s strong drug uptake capacity [25]. The HCPT injection group showed higher drug levels in the liver and spleen, whereas the Exos-HCPT group primarily accumulated in the liver, spleen, and lungs. This pattern may be attributed to the exosomal drug delivery system’s susceptibility to phagocytosis by macrophages in the liver and spleen’s reticuloendothelial system, leading to drug enrichment in these tissues [26,27,28]. Compared with the HCPT injection group, the SP94-Exos-HCPT group maintained higher drug concentrations in the liver over an extended period, suggesting enhanced drug efficacy.

## 3. Discussion

Liver cancer is the sixth most common cancer globally and the second leading cause of cancer-related deaths. HCC is frequently treated with HCPT; however, HCPT suffers from low water solubility and poor stability. To address these limitations, this study leverages the advantages of exosomes as drug carriers to construct a targeted drug delivery system for HCC. We evaluated the therapeutic efficacy of this system in vitro, as well as its pharmacokinetics and tissue distribution in animal models. The results indicate that exosomes maintain effective drug delivery properties, and the SP94-Exos-HCPT system demonstrates targeted delivery to the liver, attributed to the liver-targeting capabilities of the SP94 peptide. Compared with HCPT treatment, SP94-Exos-HCPT significantly enhanced the therapeutic effect of HCPT on HepG2, which could reduce tumor cell proliferation, inhibit tumor cell migration and invasion, promote cell apoptosis, and induce cell cycle arrest. In vitro experiments confirmed that SP94-Exos-HCPT exhibits strong targeting properties toward HepG2 cells. However, no significant differences in AUC_(0-t)_ and MRT_(0-t)_ were observed between HCPT, Exos-HCPT, and SP94-Exos-HCPT injection groups in rats, potentially due to suboptimal drug concentrations or complex metabolic activities in the biological system. Further studies, including efficacy evaluations in mouse models, are required to address these limitations. DSPE-PEG_2000_, a self-assembling phospholipid bilayer amphiphile commonly used to carry targeted ligands, such as SP94, has a hydrophobic end that can embed into exosomes. Future improvements could utilize fluorescence tracking to visualize the conjugation process. Additionally, exosomes carry various bioactive molecules, particularly miRNAs, which play a critical role in intercellular communication. Future research should employ RNA sequencing to identify miRNAs associated with liver function repair and design experimental validations.

Several recent studies have explored similar drug delivery systems. Yang et al. developed an engineered brain-targeted delivery system (ACTE) that delivers small interfering RNA towards transform growth factor-β (siTGF-β), loaded with doxorubicin (DOX) in exosomes (Ds@ACTE) [29]. The Ds@ACTE system reprograms the immunosuppressive microenvironment by downregulating TGF-β expression, thereby enhancing the chemotherapeutic effects of DOX-induced anti-tumor immune responses [29]. Zhang et al. created a doxorubicin hydrochloride polymer vesicle (PS-DOX) and demonstrated that SP94 peptide modification significantly improved the specificity, uptake, and anticancer activity of PS-DOX in liver cancer cells, effectively inhibiting orthotopic liver tumor xenografts [30]. Additionally, paclitaxel (PTX)/NCTD nanoparticles modified by tumor neovascular-targeting peptide (APRPG) have been shown to induce apoptosis through AKT and ERK signaling pathways, enhancing the efficacy of anti-liver cancer drugs [31]. It can be seen that the targeted nanomedicine delivery system can reduce the side effects of free drugs, making it a safe and effective local chemotherapy drug delivery system.

In this study, the unique structural advantages of exosomes and the targeting capability of the SP94 peptide enable SP94-Exos-HCPT to deliver drugs specifically to cancer cells while minimizing side effects on normal liver cells. Thus, the newly developed SP94-Exos-HCPT holds promise as an effective nanoparticle delivery system for the treatment of HCC.

## 4. Materials and Methods

### 4.1. Materials

HCPT (purity ≥ 98%) was purchased from McLean Biochemical Technology Co., Ltd. (Shanghai, China). DSPE-PEG2000-SP94 peptide was purchased from Feiyue Technology Co., Ltd. (Wuhan, China). Dulbecco Modified Eagle Medium (DMEM) was purchased from Gibco (New York, NY, USA). Bovine serum albumin was purchased from Meilun Bio (Dalian, China). Cell Counting Kit 8 (CCK-8) was purchased from Meilun Bio (Dalian, China). Annexin V-FITC apoptosis detection kit was purchased from Nanjing Kaigiobiology (Nanjing, China). The cell cycle assay kit was purchased from Nanjing Kaiji Biology (Nanjing, China). All solvents and other chemicals are analytical grade.

BMSC cells (mouse mesenchymal stem cell line) and HepG2 cells (human liver cancer cell line) from Yimu Biology (Xiamen, China) were prolificated in DMEM supplemented with 10% FBS and 1% penicillin. All cell types were grown in a humid environment containing 5% (*v*/*v*) CO_2_ at 37 °C. Male Sprague–Dawley rats (200 ± 20 g) and male Kunming mice (40 ± 20 g) were purchased from Guangzhou Ruge Biotechnology Co., Ltd., (Guangzhou, China). The experiments were conducted in accordance with the guidelines issued by Guangdong Pharmaceutical University.

### 4.2. Extraction and Identification of Exosomes

The supernatant of cells was collected by starvation method. First, the bone marrow mesenchymal stem cells were seeded onto culture dish, and when the growth reached about 80%, the medium was replaced with serum-free medium. The cells were then incubated for 48 h to allow exosome production. After the supernatant was collected, the exosomes were isolated by ultra-fast centrifugation [32]. The supernatant was filtered and centrifuged at 100,000× *g* for 70 min using an ultracentrifuge, followed by a second round of centrifugation. The resulting pellet was collected, resuspended in an appropriate volume of PBS, and stored at −80 °C for further use. Morphological evaluation of exosomes was performed by transmission electron microscopy. Specifically, 10 μL exosomes were added to the surface of the copper mesh, and the floating liquid was absorbed by filter paper at the edge of the copper mesh and then negatively stained with 3% phosphotungstic acid solution of equal volume for 4 min, and then absorbed by filter paper. After drying at room temperature for 30 min, the images were collected on the computer, the electron microscope accelerated voltage was adjusted to 100 kv, and the particle morphology was observed. The particle size was measured by nanoparticle tracking analyzer (NTA), and exosomal biomarkers CD9, CD63, CD81, and TSG101 were detected by Western blot analysis.

### 4.3. Preparation of Targeted Drug Delivery System

HCPT was extracted and dissolved in DMSO to prepare a stock solution, which was then diluted with PBS to a concentration of 20 μg/mL. The exosome suspension was adjusted to a concentration of 100 μg/mL in PBS, and an equal volume of the HCPT solution was added. The mixture was placed in an electroporation cuvette, and the electroporation parameters were set to 550 V. With six pulses, HCPT-loaded exosomes (Exos-HCPT) were obtained.

DSPE-PEG2000-SP94 was then modified onto the exosome membrane using the post-insertion method. Specifically, 1 mg of DSPE-PEG2000-SP94 was dissolved in PBS to create a stock solution, which was then diluted to a concentration of 5 μg/mL. This solution was co-incubated with the HCPT-loaded exosomes at a 1:1 volume ratio for 4 h. The DSPE moiety was inserted into the exosome membrane, resulting in the formation of targeted exosomes (SP94-Exos-HCPT). Finally, any free peptides were removed through ultracentrifuge.

### 4.4. In Vitro Drug Release

The drug release behavior of SP94-Exos-HCPT in vitro was investigated by dynamic dialysis method. The activated dialysis bags were filled with HCPT solution and SP94-Exos-HCPT solution, respectively, and the ends were securely tied with cotton string to prevent leakage. The dialysis bags were then immersed in a release medium containing 1% sodium dodecyl sulfate (SDS) at pH 7.4 and placed in a constant-temperature water bath shaker (37 °C, 100 rpm) to simulate the physiological environment. To mimic the tumor microenvironment, another pretreated dialysis bag containing SP94-Exos-HCPT was placed in a pH 5.8 release medium with 1% SDS and incubated in a constant temperature water bath shaker (42 °C, 100 rpm). Starting from 0 h, 4 mL of the release medium was withdrawn at specific intervals, with an equal volume of fresh release medium added to maintain constant conditions. The collected samples were filtered through a 0.45 μm filter membrane and stored in liquid vials. The HCPT content in the samples was determined by high-performance liquid chromatography (HPLC), and the cumulative release was calculated.

### 4.5. Cell Proliferation Assay

HepG2 cells were seeded into 96-well plates and divided into five groups: control group, blank exosome group, Exos-HCPT group, HCPT group, and SP94-Exos-HCPT group, whose drug concentration was 20 μg/mL. Following treatment, the 96-well plates were incubated under standard cell culture conditions. After 24 h, CCK-8 solution was added to each well, followed by incubation for an additional 30 min. The absorbance of each sample was measured using a microplate reader, and cell viability was calculated.

### 4.6. Cell Migration and Invasion Assay

In parallel, HepG2 cells were seeded into 6-well plates. The untreated group served as the control, and the same groups were established as mentioned above. After drug treatment, cells were inoculated into the upper chamber of a Transwell system, with serum-containing medium added to the lower chamber. Following 24 h of incubation, cells in the upper chamber were removed using cotton swabs, fixed, and stained. Cell motility in each group was assessed under a microscope.

For the cell invasion assay, the experimental setup was identical to the cell motility assay, with the addition of Matrigel diluted with complete medium to form an artificial basement membrane in the upper chamber of the Transwell. Treated cells were seeded into the upper chamber, and serum-containing medium was added to the lower chamber. After an appropriate incubation period, Matrigel and any non-invading cells were removed with cotton swabs. The invading cells were then fixed and stained, and their invasion rate was quantified by microscopy through image analysis and cell counting.

### 4.7. Flow Cytometry Analysis of the Cell Apoptosis and Cycle

HepG2 cells were seeded into 6-well plates and treated with blank exosomes, Exos-HCPT, HCPT, and SP94-Exos-HCPT, whose drug concentration was 20 μg/mL for 24 h. Following incubation, both floating and adherent cells were collected, washed with cold PBS, and centrifuged. The cells were resuspended in buffer, transferred to flow cytometry tubes, and incubated with Annexin V-FITC and PI for staining. The percentage of apoptotic and necrotic cells was then analyzed and quantified using flow cytometry.

For the cell cycle analysis, the same treatment and grouping conditions were applied. After 24 h of drug treatment, the cells were transferred to EP tubes. The cells were then fixed overnight in 75% pre-cooled ethanol. After fixation, the ethanol was removed by washing with pre-cooled PBS, followed by centrifugation. The cells were added with Ranase A and PI dye and stained away from light. Finally, the red fluorescence signals were detected at an excitation wavelength of 488 nm using flow cytometry, enabling cell cycle phase distribution analysis.

### 4.8. Pharmacokinetic Studies

After anesthetizing the animals with isoflurane, 0.5 mL of blood was collected from the orbital venous plexus using capillary tubes. The blood was centrifuged at 3500 rpm for 10 min to separate 100 μL of plasma. To the plasma, 200 μL of acetonitrile was added, and the mixture was vortexed for 1 min. The sample was then centrifuged at 15,000 rpm for 10 min. The supernatant was collected, evaporated to dryness under nitrogen, and the residue was dissolved in 200 μL of methanol using ultrasound. The solution was filtered through a 0.22 μm membrane and analyzed by HPLC.

Twenty-four male Sprague–Dawley rats, weighing 200 g ± 20 g, were randomly assigned to three groups: HCPT control group, Exos-HCPT group, and SP94-Exos-HCPT group, with 8 rats per group. Each group received a tail vein injection at a dose of 10 mg/kg. Blood samples were collected from the orbital venous plexus at 0.15, 1, 1.5, 2, 4, 6, 8, and 12 h post-administration. The blood was placed into centrifuge tubes containing heparin sodium and processed according to the plasma sample preparation method described above, followed by HPLC analysis.

### 4.9. Biodistribution Studies

72 male Kunming mice were randomly divided into three groups: HCPT control group, Exos-HCPT group, and SP94-Exos-HCPT group. Each group received a tail vein injection at a dose of 10 mg/kg. At 1, 4, 8, and 12 h post-administration, the mice were anesthetized with isoflurane, euthanized by cervical dislocation, and the hearts, livers, spleens, lungs, and kidneys were immediately dissected. The tissues were rinsed with normal saline to remove residual blood, blotted dry with filter paper, and weighed. Each tissue was homogenized by adding an equivalent volume of normal saline (1:1, g/mL). For tissue homogenate processing, 100 μL of the homogenate was mixed with 200 μL of acetonitrile, vortexed for 1 min, and centrifuged at 15,000 rpm for 10 min. The supernatant was collected and evaporated to dryness under nitrogen. The residue was dissolved in 200 μL of methanol using ultrasound, vortexed to mix, and filtered through a membrane. The filtrate was then transferred to a sample vial for HPLC analysis.

### 4.10. Statistical Analysis

Statistical analyses were performed with GraphPad Prism software, and the pharmacokinetic parameters were analyzed by DAS 2.0 software. Data analyses were performed with Student’s t-test and one-way analysis of variance (ANOVA). *p* < 0.05 was considered to be statistically significant.

## 5. Conclusions

In this study, we successfully developed a targeted drug delivery system, SP94-Exos-HCPT, by modifying exosomes with the SP94 peptide. In vitro experiments demonstrated that this targeted delivery system exhibits strong targeting capabilities and significantly enhances the therapeutic efficacy of HCPT. The optimized combination of an exosome-based drug delivery system with chemotherapy agents offers a novel approach to liver cancer treatment and may also serve as a valuable reference for the treatment of other tumors.

## Figures and Tables

**Table 1:** The statistical parameters of pharmacokinetics in SD rats after caudal intravenous administration.

Parameters	HCPT Group	Exos-HCPT Group	SP94-Exos-HCPT Group
AUC_0-t_ (mg/L*h)	23.6 ± 2.68	20.26 ± 3.7	23.92 ± 3.19
AUC_0-∞_ (mg/L*h)	24.3 ± 2.66	21.73 ± 3.8	31.51 ± 8
MRT_0-t_ (h)	2.3 ± 0.29	2.14 ± 0.32	2.62 ± 0.42
MRT_0-∞_ (h)	2.77 ± 0.37	3.63 ± 2.09	4.85 ± 3.08
T_1/2_ (h)	2.45 ± 0.61	4.04 ± 2.56	5.3 ± 4.73
C_max_ (mg/L)	11.68 ± 3.14	10.33 ± 1.64	12.47 ± 1.79
CL (L/h/kg)	0.42 ± 0.04	0.47 ± 0.08	0.34 ± 0.08

Abbreviations: AUC: area under the drug concentration–time curve values; MRT: mean residence time; t_1/2_: half-life time; Cmax: the maximum concentration; CL: clearance.

## Data Availability

Data are contained within the article.
